# Low serum levels of dehydroepiandrosterone sulfate and testosterone in Albanian female patients with allergic disease

**DOI:** 10.1038/s41598-021-85214-5

**Published:** 2021-03-10

**Authors:** Violeta Lokaj-Berisha, Besa Gacaferri Lumezi, Naser Berisha

**Affiliations:** 1 Institute of Physiology and Immunology, Faculty of Medicine, University of Prishtina “Hasan Prishtina”, Bulevardi i Dёshmorёve p.n, 10000 Prishtina, Kosovo; 2 Departments of Obstetrics and Gynecology, University Clinical Center of Kosovo, Prishtina, Kosovo

**Keywords:** Immunological disorders, Endocrinology

## Abstract

Evidence from several unrelated animal models and some studies conducted in humans, points to the immunomodulatory effects of androgens on various components of the immune system, especially on allergic disorders. This study evaluated the serum concentrations of sex hormones in women with allergy. For this purpose, blood samples were obtained from 78 participants in order to detect serum IgE concentrations, total testosterone, estradiol, progesterone, and DHEA-S. The majority of the subjects (54) in the study were consecutive patients with doctor-diagnosed allergic pathologies: 32 with allergic rhinitis, 10 with asthma and rhinitis, and 12 with skin allergies. In addition, 24 healthy volunteers were included in the research as the control group. The average age of the subjects was 32.54 years (SD ± 11.08 years, range between 4–59 years). All participants stated that they had not used any medical treatment to alleviate any of their symptoms prior to taking part in the research. They all underwent skin-prick tests for common aero-allergens, which was used as criterion for subject selection. Hence, the subjects were selected if they reacted positively to at least one aero-allergen. Their height and weight were measured in order to calculate the BMI. As a result, statistically significant differences between controls and allergic women in serum concentrations of androgens (testosterone, *p* = 0.0017; DHEA-S, *p* = 0.04) were found, which lead to the conclusion that the concentration of total serum testosterone and DHEA-S was lower in female patients with allergic diseases compared to controls.

## Introduction

The worldwide rising prevalence of allergic diseases that have been registered in the World Health Organization (WHO) statistics is documented by several population-based birth cohort studies. A steady increase has occurred with about 30–40% of the world population now being affected by one or more allergic conditions^1^. Although, the reasons for growing allergy prevalence remain largely unclear, Multi-center Allergy Studies are crucial in determining genetic, environmental, life-style related, and protective factors for better prevention strategies^2^.

There are many lines of evidence about the relationship between allergic disease and sex hormones; however, the reported data and subsequent conclusions often conflict. According to research conducted in several countries, there is a predominance of allergic disease in males at pre-puberty age and this reverses to females to post-puberty, suggesting that sex hormones may play a significant role in the expression of atopic allergies, separate from their effects on reproduction and sexual differentiation^3^.

In women, the ovaries secrete the female sex hormones but adrenal glands are also important for the secretion of major androgens^4^. In addition to testosterone, the main androgen secreted in women by adrenal glands and ovaries is dehydroepiandrosterone (DHEA) and its sulfated derivative, dehydroepiandrosterone sulfate (DHEA-S), which is a better analyte to test than DHEA itself due to its longer half-life and greater abundance. Although their specific biological role is not completely understood, DHEA and DHEA-S (DHEA/S) are weak androgens that act as precursors for biosynthesis of sex hormones^5^.

Testosterone and DHEA/S may function as immunomodulators, especially in autoimmunity and allergic disorders^6,7^. It has been hypothesized that DHEA/S may antagonize the production of TH2 cytokines, promoting the shift in the TH1/TH2 balance towards TH1-dominant immunity. On the contrary, low DHEA/S levels may up regulate TH2 cytokines^8,9^. Numerous studies have demonstrated the enhancing effect of DHEA/S at the level of IL-2 mRNA on CD4 + T cells, suggesting that DHEA-S supplementation could bring some therapeutic benefits to patients with respiratory allergies^10^. DHEA/S is a precursor molecule for sex hormones, and its secretion varies according to age, with the highest levels in women in their 30 s and lowest in very old age^11^. However, circulating levels of DHEA-S can also be very low in acute inflammatory reactions (sepsis) and in patients with chronic autoimmune diseases (rheumatoid arthritis and systemic lupus erythematosus), revealing its relevant anti-inflammatory properties^12–14^.

Interestingly, some human models demonstrated that DHEA-S decreases the production of TH2 cytokines (IL-4 and possibly IL-5), suggesting a potential regulatory function on IgE production. It was clearly demonstrated that DHEA-S administered in an experimental murine model of airway inflammation, could lower the production of TH2 cytokines and also suppress the production of IgE and eosinophilic inflammation^15^. Consequently, DHEA-S could be an endogenous modulator of allergic reactions in humans^16^.

Furthermore, previous studies in animals, and cell-culture experiments have established that testosterone has anti-inflammatory properties, suggesting a possible role of the male sex hormone in the immune response. Male patients with hypotestosteronemia demonstrated significant improvement in their allergy symptoms, along with improvement in well-being, energy levels, and overall health when given supplementary testosterone, indicating that testosterone deficiency could reduce the immune response in men^17^. In other studies, however, the testosterone levels were reported to be lower in allergic patients treated with glucocorticoids compared to those who were not treated with such drugs^18^. On the other hand, data related to testosterone levels and their role in the immune system of women is scanty at best. In a study related to testosterone and immunity conducted at Jena University in Germany, researchers found that immune cells isolated from men had less active phospholipase D than cells from females and that the enzyme activity in female immune cells decreased after testosterone treatment^19^. In an attempt to clarify the gender-specific role of androgens in allergic disease, the present study was conducted to investigate if there are significant differences in the serum concentrations of total testosterone and DHEA-S in allergic women patients compared to healthy controls.

## Results

Among the participants in the research, the total of 78 women, the prevalence of allergic diseases in the research group was: 12.82% with asthma and rhinitis, 41.02% with rhinitis and 15.38% with skin allergies (Table 1). The largest percentage of subjects was within the age group 20–29 and 30–39 years respectively, with an average age of 33.1 years. Sensitization to house dust mites had the highest prevalence among all the participants at 51%, followed by sensitization to pollens at 31.7%.

**Table 1:** Age, BMI and serum concentrations of total testosterone and DHEA-S in allergic patients and controls.

Analyzed variables	Control group n = 24	A/Rh group n = 10	Derm/Urtic group n = 12	Rh group n = 32	P-value
Age (years) Mean SD	32.10 (10.80)	30.00 (13.40)	40.00 (9.10)	30.90 (10.40)	0.03
BMI (kg/m^2^) Median, range	24.00 (16.7–36.2)	21.80 (18–28)	27.40 (20–42)	23.00 (15.8–32.8)	0.999
DHEA-S (μmol/L) Median, range	6.55 3.00–16.0	5.05 2.70–10.90	6.00 3.10–9.70	5.30 0.65–13.50	0.032
**DHEA-S (μmol/L)**					
< 0.81	–	–	1 (8.3%)	1 (3.1%)	
0.81–9.00	19 (79.2%)	7 (70.0%)	10 (83.3%)	26 (81.3%)	
> 9.00	5 (20.8%)	3 (30.0%)	1 (8.3%)	5 (15.6%)	
Testosterone (nmol/L) Median, range	2.67 1.52–4.83	1.70 0.74–3.60	2.46 0.65–3.30	2.20 0.40–5.40	0.0017
**Testosterone (nmol/L)**					
0.24–1.03	–	2 (20.0%)	3 (25.0%)	5 (15.6%)	
1.04–1.83	3 (12.5%)	4 (40.0%)	1 (8.3%)	7 (21.9%)	
1.84–2.60	9 (37.5%)	–	5 (41.7%)	10 (31.3%)	
> 2.60	12 (50%)	4 (40.0%)	3 (25.0%)	11 (34.4%)	

n—number of subjects; DHEA-S—dehydroepiandrosterone sulfate; Rh—allergic rhinitis; A/Rh—allergic asthma with rhinitis; Derm/urtic—allergic dermatitis/acute urticaria; BMI—body mass index (normal = 18.5–24.9 kg/m^2^, overweight = 25–29.9 kg/m^2^, obese > 30 kg/m^2^).

The morning serum concentration of testosterone, DHEA-S, and IgE showed a statistically significant difference between the investigated groups (p < 0.05), unlike the concentration of estradiol or progesterone, which showed no significant difference (p > 0.05).

### Serum testosterone levels

Compared to the reference values for normal serum testosterone concentration (0.24–2.6 nmol / L), it was found that 40% of patients with allergic asthma + rhinitis (A + Rh) had serum testosterone concentrations above 2.6 nmol / L, followed by 34.4% of patients with allergic rhinitis, and 25% of patients with allergic skin disease (acute allergic dermatitis or acute urticaria) (Table 1). Moreover, 50% of the controls had testosterone levels above the normal upper limit (58% of them were atopic) (Table 2). However, the average serum testosterone concentration was significantly lower in patients with allergic disease compared to controls, according to Mann Whitney test (p = 0.0017). These differences were especially marked between the healthy control group and patients with A + Rh (p = 0.024) respectively, with Rh (p = 0.01278), but not between the skin allergy group and the control group (p = 0.057). Particularly high levels of testosterone in healthy controls were found: 3.9 nmol / L in subjects with positive SPT compared to 2.6 nmol / L of those with negative SPT (Table 2).

**Table 2:** Relationship between age, BMI, serum testosterone and DHEA-S concentration in skin-prick-tested healthy subjects.

Skin prick test	Total (controls) n = 24		P value
	Positive	Negative	
Number	9	15	P = 0.192
Age (years) Mean, SD	28.3 (11.1)	34.3 (10.3)	
BMI (kg/m2) Median, range	25 (17.2–36.2)	23.2 (16.7–26)	P = 0.235
N (%)			
Normal	4 (44.4)	11 (73.3)	
Overweight	4 (44.4)	4 (26.7)	
Obese	1 (11.2)	–	
DHEA-S (μmol/L) Median, range	8.0 (4.3–16)	6.0 (3.0–13.3)	P = 0.330
**DHEA-S (μmol/L)**			
N (%)			
< 0.81	–	–	
0.81–9.0	6 (66.7)	13 (86.7)	
> 9.0	3 (33.3)	2 (13.3)	
Testosterone (nmol/L) Median, range	3.9 (1.6–4.4)	2.6 (1.5–4.8)	P = 0.108
**Testosterone (nmol/L)**			
N (%)			
0.24–1.03	–	–	
1.04–1.83	1 (11.1)	2 (13.3)	
1.84–2.6	1 (11.1)	8 (53.3)	
> 2.6	7 (77.8)	5 (33.3)	

n—number of subjects; DHEA-S—dehydroepiandrosterone sulfate; Rh—allergic rhinitis; A/Rh—allergic asthma with rhinitis; Derm/urtic—allergic dermatitis/acute urticaria; BMI—body mass index (normal = 18.5–24.9 kg/m^2^, overweight = 25–29.9 kg/m^2^, obese > 30 kg/m^2^).

### Serum DHEA-S levels

Statistically significant differences in DHEA-S concentration among the investigated groups were also found: (*p* < 0.05). Using Dun’s multiple comparison test the difference between the allergic rhinitis and the control groups was also found (*p* < 0.01) (Table 1).

### Estradiol and Progesterone

Based on the results of the Kruskal Wallis test, there were no significant differences in estradiol (*p* = *0.24*) or progesterone (*p* = *0.29*) levels between the study and control groups, regardless of the stages of the menstrual cycle. In addition, progesterone to estradiol ratio, as an essential part of female hormone balance assessment, was within the normal range of 28–74 (64 for rhinitis group, 37 for asthma + rhinitis, 42 for skin allergy and 61 for the control group) (Table 3).

**Table 3:** Serum levels and the ratio of Progesterone to Estradiol in patients and controls according to phases of menstrual cycle.

Analyzed hormones		Control group n = 24	A/Rh group n = 10	Derm/urtic group n = 12	Rh group n = 32	P-value
Progesterone (nmol/L) Median (range)	Folicular (0.19–4.0)	1.165 (0.42–3.63)	1.64 (0.7–3.5)	1.07 (0.51–3.7)	2.0 (0.3–4.5)	.609
	Ovulation (0.25–3.82)	2.8 (0.4–26.4)	4.9 (0.6–38)	6.7 (0.4–62)	7.0 (0.4–34.5)	.786
	Luteal (7.95–79.5)	29.16 (0.4–42)	20.0 (1.5–50.8)	21.0 (1.6–41.9)	25.7 (0.5–63)	.773
Estradiol (pmol/L) Median (range)	Folicular (210–833)	310.55 (106.9–916.6)	276.2 (178.9–415.6)	304.8 (156.9–681.6)	320.5 (114–960)	**.**89
	Ovulation (460–1750)	556 (121.8–1305.6)	382.5 (175.0–740.0)	501.0 (127.0–1430.6)	315.8 (80.0–1292.2)	.175
	Luteal (283–1020)	446.6 (114.1–890.0)	480.0 (81.0–2400)	451.4 (180.0–1600)	417.1 (86.0–1077.8)	.970
Pg/E ratio (Luteal phase)		61.5	37.0	41.7	64.4	
Pg/E ratio (of Means)		79.54	56.7	34.01	72.73	

n—number of subjects; Pg—progesterone; E—estradiol; Rh—allergic rhinitis; A/Rh—allergic asthma with rhinitis; Derm/urtic -allergic dermatitis/acute urticaria.

### Serum IgE levels

The total serum IgE in the allergic group was significantly higher than in the control group according to the Kruskal–Wallis test (*p* = 0.012).

### BMI

Although there were no statistically significant differences among the groups for BMI, patients with skin allergies and healthy controls with positive skin prick test tended to be overweight (BMI = 25.6 and BMI = 25 respectively) (Table 1).

### Correlation

Considering the fact that hormone levels change with age, there was a weak negative correlation between age and testosterone level (*r* = *-0.26*), as well as age and DHEA-S (*r* = *-0.39*) in all participants. A moderate positive correlation between testosterone and DHEA-S (*r* = *0.46*) was also observed in all participants except for the group with skin allergy (*p* = *-0.05*).

**Figure 1:**
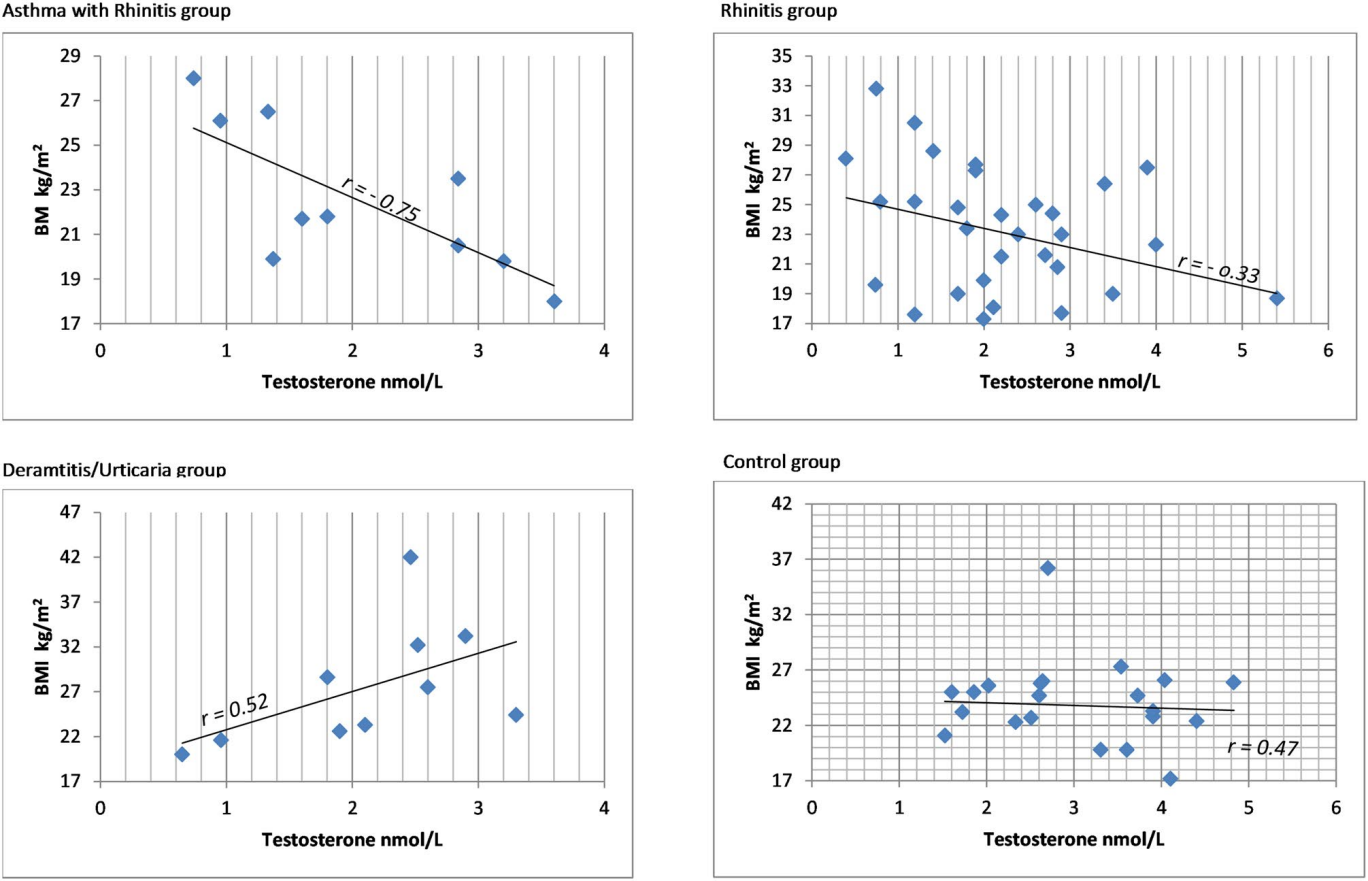
The relation between BMI and serum testosterone levels in females with allergic diseases and controls. A—patients with Asthma; A&R—patients with Asthma and Rhinitis; BMI-body mass index; *r*—the correlation coefficient.

Furthermore, there was a strong negative correlation between BMI and testosterone levels in patients with asthma + rhinitis (*r* = *-0.75*) and weak negative correlation in patients with rhinitis (r = -0.33). However, moderate positive correlation in patients with skin allergies (r = 0.52), as well as in controls (r = 0.47) was found (Fig. 1). Nonetheless, regression analyses showed that these correlations were not significant.

**Figure 2:**
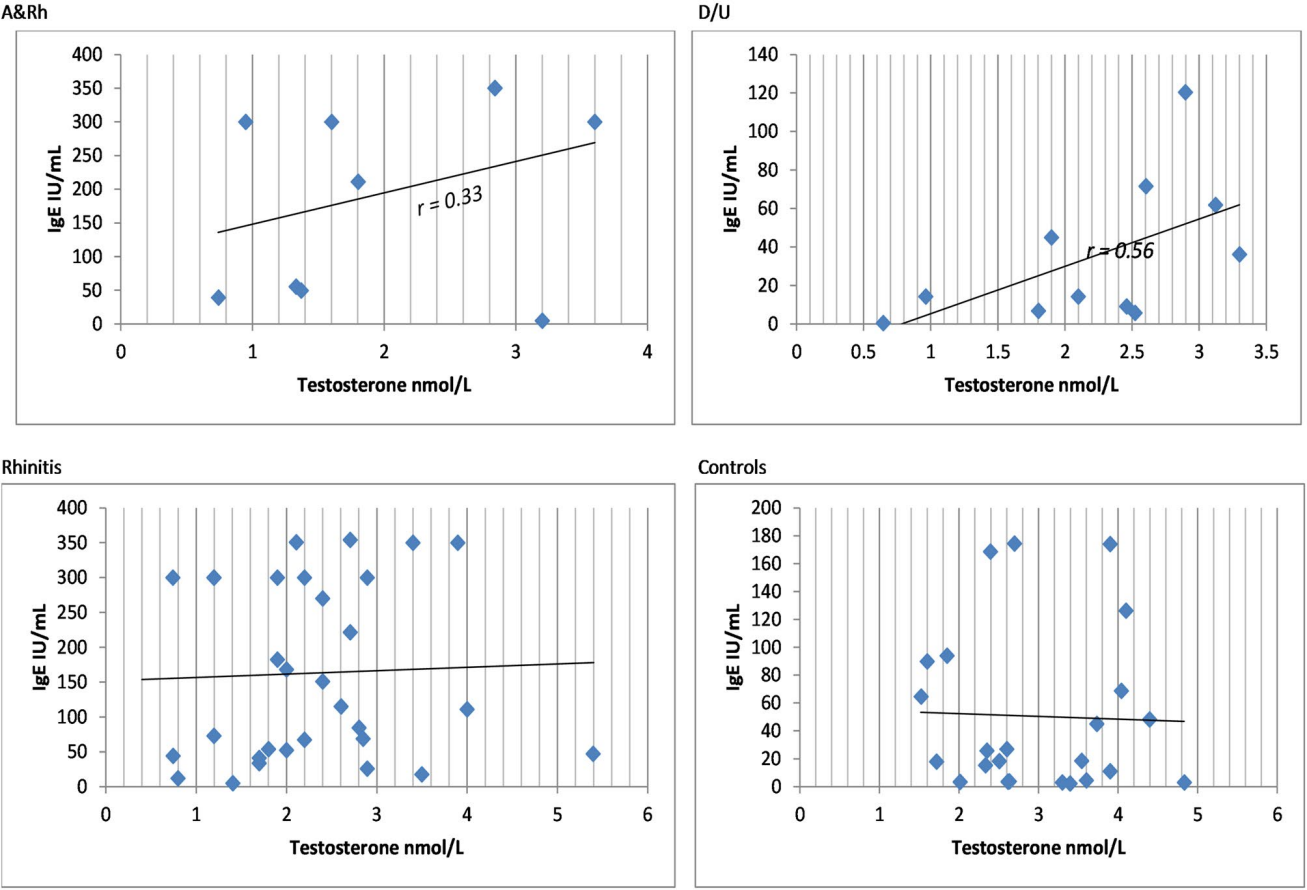
Positive correlation between total IgE levels and serum testosterone concentrations in female patients with allergies. IgE- immunoglobulin E; *r*—correlation coefficient.

Moreover, there was no correlation found between levels of DHEA-S and IgE or between DHEA-S and BMI, while only a weak to moderate correlation between IgE and testosterone in patients with asthma + rhinitis, or dermatitis/urticaria was recorded (Fig. 2).

## Discussion and conclusion

Considering that allergic disorders are in part a consequence of Th1/Th2 imbalance and, along with autoimmune diseases, in most cases females are more prone to them than males, there have been some very interesting reports about the role of sex hormones, particularly testosterone and DHEA-S, in regulating T cell proliferation and differentiation^4,10,16,19,20^. In general, male sex hormones suppress allergic responses whereas female ones aggravate them; therefore, low levels of testosterone or androgens are expected in male allergic patients and high levels of estrogen and progesterone in female allergic patients. However, they both have adrenal glands that produce the weak androgens, DHEA and its sulfated form DHEA-S, which have recently been implicated as immunomodulators^4,5,7,8,21^. The results are not entirely consistent, however, since some laboratories showed no significant differences in circulating concentrations of DHEA-S and total testosterone in allergic rhinitis and mild asthma, while others reported low levels of testosterone in allergic patients treated with corticosteroids^5,17,22,23^.

Given that in normal fertile women, serum testosterone levels can be as low as those measured in pubertal children, high concentration of testosterone in healthy control subgroup with positive skin reaction to aeroallergens, could be attributed to their high BMI (median 25 kg/m^2^ range 17.2–36.2), in contrast to subjects with asthma + rhinitis who had the lowest BMI (median 21.8 kg/m^2^ range 18–28) and also showed the lowest concentration of testosterone. Nevertheless, statistical analysis did not support a significant correlation between BMI and testosterone levels. This is further underlined by the fact that subjects with skin allergies, rhinitis, and those in the control group without skin reaction to allergens, had normal to high BMI values. Nonetheless, their testosterone levels were within the normal range (median BMI: 27.4 kg/m^2^, 23.1 kg/m^2^ and 23.2 kg/m^2^ and testosterone levels 2.46, 2.2 and 2.6 nmol/L respectively (Table 1). The idea that high BMI is not always associated with a significant increase in serum testosterone levels, as had been reported in some studies^24,25^ is also supported by our data.

Furthermore, a decline in testosterone that has been observed with growing age, does not match our evidence since older skin allergy sufferers (Mean age 40 years) had the upper normal testosterone levels (2.6 nmol/L) (Table 1), and especially it does not correlate with the observation that atopic controls with an average age of 29 years had a median testosterone level of 3.9 nmol/L (Table 2)^26^. Consequently, factors other than age and BMI should be considered for patients with allergies and especially for healthy atopic women when evaluating testosterone concentrations. As recently reported by Pergola et al., treatment of immune cells from female patients with testosterone resulted in reduced activity of phospholipase D. Consequently, it may be assumed that the high levels of testosterone observed in our study in the serum of healthy atopic subjects may in part be responsible for the absence of clinical symptoms, while, on the other hand, low values in the research group may be the cause of their occurrence^19^. However, since phospholipase D was not measured in our participants, a cause-effect relationship cannot be established.

It is suggested that renal excretory capacity for DHEA-S (and OH-progesterone and progesterone) is significantly reduced in the elderly due to changes in the zona reticularis of the adrenal cortex^27,28^. Low levels of DHEA-S could be attributed to old age; however, this does not hold true in our patients with acute allergic dermatitis/acute urticaria where the average age of the subjects was 40 and the average DHEA-S level was 6.0 μmol / L (Table 2). In addition, normal levels of estradiol and progesterone at all stages of the menstrual cycle, accompanied by their normal ratio, regardless of the age of the participants, do not support the above-mentioned statement. It can be assumed that high levels of androgens in the atopic healthy group inhibited the clinical development of allergic symptoms. However, androgens seem to act as a counterbalance to estrogen in their immunological effects, while the immunomodulatory effects of testosterone in women still remain to be clarified despite their recognized anti-inflammatory action in men^29^.

Our observations raise a question: are these lowered DHEA-S and testosterone levels indicative of a possible causal relationship between endocrine and allergic diseases, or is the co-occurrence merely incidental? There is some evidence to link them. An overlap between metabolic and endocrine disorders has been proposed in the context of asthma, but our data show a link in the context of rhinitis. Overall, the lack of agreement among recent investigations suggests that there is a strong need for further studies on the role of androgens in women. The relatively small sample size in our research may have been inadequate to allow effective conclusions to be drawn. Consequently, further studies are required to elucidate the molecular mechanisms of androgen-induced immunosuppression, leading to the identification of novel therapeutic targets for immune disorders. In conclusion, our results suggest that female patients with respiratory allergies have lower concentrations of total testosterone and DHEA-S compared to healthy subjects. Clinical experience has shown that a substantial number of rhinitis patients exhibit overt atopic manifestations and lowered DHEA-S levels. There is published evidence to support a link between allergy and endocrine disorders, which makes it worthwhile to assess rhinitis patients for androgen levels.

## Patients and methods

### Subjects

Fifty-four (54) female subjects with doctor-diagnosed allergic disease and twenty-four (24) healthy volunteers were recruited for this study. The study group participants were consecutive patients at the allergy outpatient service of the University Clinical Center in Prishtina. All subjects affirmed that they had not used any medication that might influence hormone levels, particularly corticosteroids, for the treatment of allergy symptoms, or other health issues, prior to the commencement of this trial. All of the subjects had regular menstrual cycles and did not use contraceptives before the study.

After having been evaluated for their allergy symptoms and diagnosed with any respiratory or skin diseases, informed written consent from the subjects, as well as parental consent was for participants younger than 16 years of age was obtained. Each subject filled out a questionnaire regarding their demographic data, family history of atopy, respiratory symptoms and smoking history. Heights were measured with a stadiometer and weights by electronic scale. Based on these BMIs were calculated—body weight in kilograms divided by the square of the height in meters (kg/m^2^). The body mass indices were classified as low (BMI, < 18.5 kg/m^2^), normal (18.5–24.9 kg/m^2^), overweight (25–29.9 kg/m^2^), and obese (BMI, ≥ 30 kg/m^2^) in accordance with the World Health Organization (WHO), the US Preventive Services Task Force and the International Obesity Task Force.

Healthy volunteers who had never had symptoms of respiratory allergies in their entire life (coughing, sneezing, itching, and nasal discharge) were selected as controls. The average age of the participants in the study was 32.54 years (SD ± 11.08 years), ranging from 14 to 59 years (32.7 SD ± 11.31 of the study group and 32.1 years SD ± 10.8 of controls, respectively). The research project protocol was approved by the Faculty of Medicine Ethics Committee, University of Prishtina “Hasan Prishtina”, Republic of Kosovo (reference number 3126/17.10.2011), and all experiments were performed in accordance with the relevant guidelines and regulations.

### Exclusion criteria

Patients with secondary ovarian failure and oophorectomy were excluded from the study.

### Investigations

#### Blood sampling

Venous blood (5 mL) was drawn at 9:00 a.m. on the seventh day of the menstrual cycle and used to measure total IgE, total testosterone, estradiol, progesterone and DHEA-S. Two other blood samples were taken on the fourteenth and the twenty-first days of the menstrual cycle to measure estradiol and progesterone through the whole cycle. Blood samples were allowed to clot at room temperature and centrifuged at 1200 × g for 10 min to separate the serum. Aliquots were stored at -80℃ until use. The laboratory of Endocrinology, Institute of Physiology, University Clinic Centre (UCC) Prishtina performed the assays for total IgE, total testosterone, estradiol, progesterone and DHEA-S levels using RIA (Beckman Coulter Immunotech, France). The reference ranges were 210–1750 pmol/L for estradiol (210–883 the follicular phase, 460–1750 the ovulation, and 283–1020 the luteinizing phase); 0.19–79.5 nmol/L for progesterone (0.19–4.0 the follicular phase, 0.25–3.8 the ovulation, and 7.95–79.5 the luteinizing phase); 0.24–2.6 nmol/L for total testosterone; 0.81–9 μmol/L for DHEA-S, and 183 IU/mL for IgE levels.

### Skin prick test (SPT)

All participants were tested for panel allergens by SPT (G aeroallergens, Allergopharma, Reinbeck, Germany). Histamine, 1 mg/ml was applied as positive control and saline as negative. Allergenic pollen extracts included grasses (grasses/cereals, grasses, rye), trees (alder, hazel, birch, beech) and weeds (mugwort, E. plantain). House dust mite extract (*D. pter.* and *D. far*.), animal dander (cat, dog, golden hamster and guinea pig) and molds (*Alternaria, Aspergillus, Cladosporium* and *Penicillium notatum*) were also tested. Allergens were applied to the forearm using a separate sterile lancet for each allergen. After fifteen minutes, the diameter of the skin reaction was recorded and considered positive if the weal/flare diameter was greater than 3 mm. All of the study patients were allergic to at least one allergen, and some participants from the control group also reacted positively.

### Statistical analyses

Data are presented as median and range. For determining the significance of differences among the groups, the Kruskal–Wallis variance analysis and Dun’s multiple comparison tests were used.

On the other hand, Fisher’s exact test and Mann–Whitney test were used to assess the differences between two groups of healthy controls (positive or negative to SPT). *P* values < 0.05 were considered significant.

### Ethics declarations

The research project protocol was approved by the Faculty of Medicine Ethics Committee (reference number 3126/17.10.2011), University of Prishtina “Hasan Prishtina”, Republic of Kosovo and all experiments were performed in accordance with the relevant guidelines and regulations. We also declare that the informed written consent was obtained from all participant and/or their legal guardians.

## Data Availability

The datasets generated and/or analyzed during the current study are available and can be obtained from the corresponding author upon a justifiable request.
